# Assessing the Use of Wrist-Worn Devices in Patients With Heart Failure: Feasibility Study

**DOI:** 10.2196/cardio.8301

**Published:** 2017-12-19

**Authors:** Yasbanoo Moayedi, Raghad Abdulmajeed, Juan Duero Posada, Farid Foroutan, Ana Carolina Alba, Joseph Cafazzo, Heather Joan Ross

**Affiliations:** ^1^ Ted Rogers Centre of Excellence in Heart Function University Health Network Toronto, ON Canada; ^2^ Centre for Global eHealth Innovation Techna Institute University Health Network Toronto, ON Canada

**Keywords:** MeSH: exercise physiology, heart rate tracker, wrist worn devices, Fitbit, Apple watch, heart failure, steps

## Abstract

**Background:**

Exercise capacity and raised heart rate (HR) are important prognostic markers in patients with heart failure (HF). There has been significant interest in wrist-worn devices that track activity and HR.

**Objective:**

We aimed to assess the feasibility and accuracy of HR and activity tracking of the Fitbit and Apple Watch.

**Methods:**

We conducted a two-phase study assessing the accuracy of HR by Apple Watch and Fitbit in healthy participants. In Phase 1, 10 healthy individuals wore a Fitbit, an Apple Watch, and a GE SEER Light 5-electrode Holter monitor while exercising on a cycle ergometer with a 10-watt step ramp protocol from 0-100 watts. In Phase 2, 10 patients with HF and New York Heart Association (NYHA) Class II-III symptoms wore wrist devices for 14 days to capture overall step count/exercise levels.

**Results:**

Recorded HR by both wrist-worn devices had the best agreement with Holter readings at a workload of 60-100 watts when the rate of change of HR is less dynamic. Fitbit recorded a mean 8866 steps/day for NYHA II patients versus 4845 steps/day for NYHA III patients (*P*=.04). In contrast, Apple Watch recorded a mean 7027 steps/day for NYHA II patients and 4187 steps/day for NYHA III patients (*P*=.08).

**Conclusions:**

Both wrist-based devices are best suited for static HR rate measurements. In an outpatient setting, these devices may be adequate for average HR in patients with HF. When assessing exercise capacity, the Fitbit better differentiated patients with NYHA II versus NYHA III by the total number of steps recorded. This exploratory study indicates that these wrist-worn devices show promise in prognostication of HF in the continuous monitoring of outpatients.

## Introduction

Exercise capacity and raised heart rate (HR) are important prognostic markers in patients with heart failure (HF) [1,2]. In clinic, we rely on patients’ self-reported exercise capacity and classify their symptoms based on the New York Heart Association (NYHA) scale. Although widely used, this classification is subjective and poorly reproducible [2]. Furthermore, clinicians are exposed to only a snapshot of the patients’ HR in the ambulatory setting. There has been significant growth in wrist-worn fitness devices that track activity and HR [3]. These wearable devices use infrared and green light emitting diodes to track HR using the photoplethysmography (PPG) method [4]. The aim of this study was to validate the accuracy of HR monitoring using Fitbit and Apple Watch at rest and during structured cardiopulmonary exercise testing in healthy individuals and to then examine the relationship of physical activity in patients with HF.

## Methods

We conducted a two-phase study assessing the accuracy of HR using Apple Watch and Fitbit wrist-based devices in healthy participants and then as continuous HR monitoring in patients with HF. In Phase 1, 10 healthy individuals wore a Fitbit, an Apple Watch, and a GE SEER Light 5-electrode Holter monitor while exercising on a cycle ergometer with a 10-watt step ramp protocol. During the first 60 seconds of the test, the workload was set to 0 watts and followed by increments of 10 watts with a maximum workload of 100 watts. In the recovery period, the workload was decreased to 10 watts. In Phase 1, two participants were excluded as data from one device could not be recorded. In Phase 2, 10 patients with HF with NYHA Class II-III symptoms wore both wrist devices for 14 days to capture overall step count/exercise levels. Two patients were excluded due to incomplete data recorded.

For Phase 1, we calculated a single measures intraclass correlation (ICC) and a 95% confidence interval, specific to each exercise workload, between HR measured by Holter (gold standard) and HR measured by the Fitbit and Apple Watch devices. The mean ICC and 95% confidence interval for each device against the Holter as the gold standard was plotted against exercise workload.

For Phase 2, we used a Kruskal-Wallis rank test to compare the mean number of steps, calculated by either device, between patients in NYHA Class II or III. We used STATA 13.1 and SPSS 22 statistical packages for our statistical analysis. The study protocol was approved by the University Health Network Research Ethics Board.

## Results

**Figure 1:**
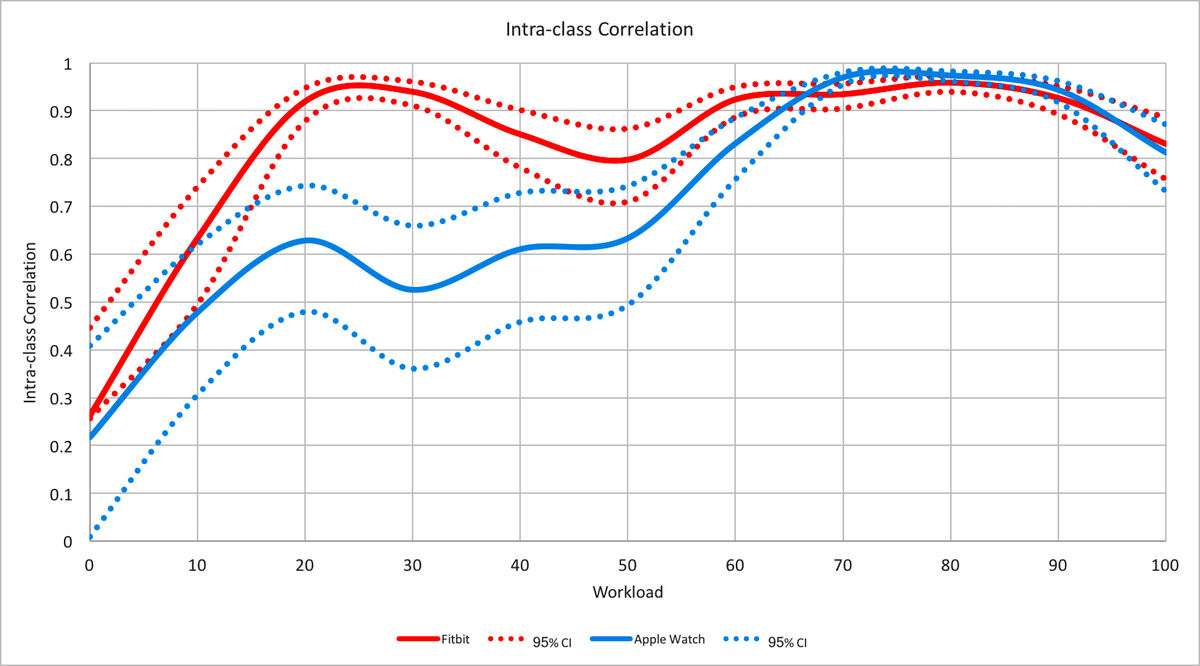
Intraclass correlation curves (solid) with 95% confidence intervals (dotted) for workload comparing Fitbit to Holter and Apple Watch to Holter in healthy individuals.

Recorded HR by both devices was not significantly related with the Holter HR at rest (Fitbit ICC=.263, 95% CI 0.257-0.447, Apple Watch ICC=.218, 95% CI 0.010-0.408). However, with cycle ergometer workloads of 60-100 watts, both devices had stronger agreement with the Holter HR (Figure 1).

**Figure 2:**
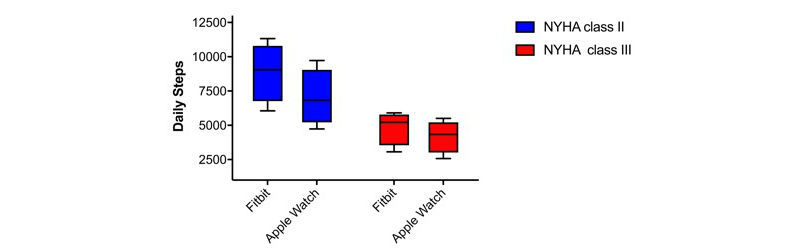
Total daily steps recorded (with 95% confidence interval) by Fitbit and Apple watch over 14 days for patients with New York Heart Association (NYHA) Class II and III symptoms .

Table 1 shows the baseline demographics of the patients included in Phase 2. Patients were predominantly male (5/8, 63%), with an average age of 58 years and ischemic cardiomyopathy (5/8, 63%). All patients were on guideline-directed medical therapy including a betablocker and either an angiotensin-converting enzyme (ACE) inhibitor or angiotensin-receptor blocker (ARB) when indicated. As shown in Figure 2, Fitbit recorded a mean 8866 steps/day for NYHA II patients versus 4845 steps/day for NYHA III patients (*P*=.04). In contrast, Apple Watch recorded a mean 7027 steps/day for NYHA II patients and 4187 steps/day for NYHA III patients (*P*=.08).

**Table 1:** Demographics and baseline data.

Number	Age (years)	Gender	LVEF^a^, %	Etiology of HF^b^	NYHA^c^ class	Medications^d^		
						Betablocker	Amiodarone	Other
1	67	Male	40	Ischemic	3	Bisoprolol 2.5 mg	None	Candesartan 8 mg
2	68	Male	18	Ischemic	2	Bisoprolol 10 mg	200	Irbesartan 300 mg
3	63	Male	25	Ischemic	3	Bisoprolol 10 mg	None	Perindopril 8 mg
4	61	Female	27	Non-ischemic	2	Bisoprolol 10 mg	None	Perindopril 4 mg
5	52	Male	25	Ischemic	2	Bisoprolol 10 mg	None	Perindopril 8 mg
6	57	Female	27	Non-ischemic	3	Carvedilol 25 mg	None	Ramipril 2.5 mg
7	58	Female	60	Familial	2	None	None	None
8	35	Male	33	Hypertrophic	3	Carvedilol 50 mg	None	Ramipril 10 mg

^a^LVEF: left ventricular ejection fraction.

^b^HF: heart failure.

^c^NYHA: New York Heart Association.

^d^Drug doses are total daily dose.

## Discussion

### Principal Findings

In this study, both PPG-based monitors had difficulty predicting HR when compared to a 5-lead electrode electrocardiogram (ECG) Holter as the gold standard in a cardiopulmonary study where HR is expected to change quickly over a 10-minute period. A recent study by Wang et al reported similar variability among four popular wrist-worn devices in relation to standard ECG limb leads and a Polar H7 chest strap monitor [5]. The HR underestimation was due to the inherent limitation of PPG that requires a longer settling time processing and averaging to eliminate optical and motion artifact. As a result, PPG monitors underestimated HR and showed poor correlation until the latter stages of the ramp study where HR would tend to saturate and the settling time was sufficient for the PPG HR estimation to compare favorably to the ECG gold standard. Although this indicates that PPG is less suited for dynamic HR measurements, in an outpatient monitoring context, PPG may be suitable for long-term static measurement of HR over long periods of time where settling time would not be an issue [6].

### Strengths and Limitations

When assessing exercise capacity, the Fitbit better differentiated patients with NYHA II versus III by the total number of steps recorded. The limitations in this study include the small sample size; larger studies will be needed to confirm these findings. This is the first study in the literature that suggests the possibility of better classifying patients, quantitatively, and potentially remotely, rather than the current practice of determining class through self-reported symptoms with its inherent limitations. Reductions in daily step counts may herald the onset of progressive NYHA symptoms alerting physicians to assess patients in a timely manner.

### Conclusion

This exploratory study indicates that wrist-worn devices show promise in prognostication of HF in the continuous monitoring of outpatients, but they require further validation.

## Abbreviations

ACE: angiotensin-converting enzyme
ARB: angiotensin-receptor blocker
ECG: electrocardiogram
HF: heart failure
HR: heart rate
ICC: intraclass correlation
NYHA: New York Heart Association
PPG: photoplethysmography
