# Correction: Assessing the Use of Wrist-Worn Devices in Patients with Heart Failure: Feasibility Study

**DOI:** 10.2196/10149

**Published:** 2018-05-04

**Authors:** Yasbanoo Moayedi, Raghad Abdulmajeed, Juan Duero Posada, Farid Foroutan, Ana Carolina Alba, Joseph Cafazzo, Heather Joan Ross

**Affiliations:** ^1^ Ted Rogers Centre of Excellence in Heart Function University Health Network Toronto, ON Canada; ^2^ Centre for Global eHealth Innovation Techna Institute University Health Network Toronto, ON Canada

The authors of the paper “Assessing the Use of Wrist-Worn Devices in Patients with Heart Failure: Feasibility Study” (Cardio JMIR 2017;1 (2): July-Dec) made a mistake by including a patient who had heart failure with preserved ejection fraction. This finding was just brought to the authors’ attention. They apologize for this oversight but have taken all measures to ensure that correct data is displayed in the article.

In the Introduction section, the following has been removed from the end of the final sentence:

...with reduced ejection fraction (HFrEF; ejection fraction <40%) anda NYHA Class II and III, as measured by daily steps by these two devices.

In the Methods section, the mention of “HFrEF” has been changed to “HF”. “HFrEF” has also been removed from the paper's Abbreviations list.

In the Results section, the second and third sentences of the paragraph beginning with “Table 1...” has been changed to the following:

Patients were predominantly male (5/8, 63%), with an average age of 58 years and ischemic cardiomyopathy (5/8, 63%). All patients were on guideline-directed medical therapy including a betablocker and either an angiotensin-converting enzyme (ACE) inhibitor or angiotensin-receptor blocker (ARB) when indicated.

Specifically, the text which was previously “(6/8, 75%)” now reads “(5/8, 63%)”. The average age was “57” and is now “58”, and the phrase “when indicated” has been added to the end of the latter sentence.

The caption for Table 1 has been shortened from “Demographics and baseline data of patients included in phase 2 of the study” to “Demographics and baseline data”. In Table 1 itself, data for Patient 7 has been changed under the following columns:

**Age (years):** “51” changed to “58”

**Gender:** “Male” changed to “Female”

**LVEF^a^, %:** “35” changed to “60”

**Etiology of HF^b^:** “Ischemic” changed to “Familial”

**Medications^d^, Betablocker:** “Carvedilol 12.5 mg” changed to “None”

**Medications^d^, Other:** “Ramipril 10 mg” changed to “None”

The updated version of Table 1 is available below.

The corrected article will appear in the online version of the paper on the JMIR website on May 4, 2018, together with the publication of this correction notice. Because this was made after submission to PubMed, Pubmed Central, and other full-text repositories, the corrected article also has been re-submitted to those repositories.

**Table 1:** Demographics and baseline data.

Number	Age (years)	Gender	LVEF^a^, %	Etiology of HF^b^	NYHA^c^ class	Medications^d^		
						Betablocker	Amiodarone	Other
1	67	Male	40	Ischemic	3	Bisoprolol 2.5 mg	None	Candesartan 8 mg
2	68	Male	18	Ischemic	2	Bisoprolol 10 mg	200	Irbesartan 300 mg
3	63	Male	25	Ischemic	3	Bisoprolol 10 mg	None	Perindopril 8 mg
4	61	Female	27	Non-ischemic	2	Bisoprolol 10 mg	None	Perindopril 4 mg
5	52	Male	25	Ischemic	2	Bisoprolol 10 mg	None	Perindopril 8 mg
6	57	Female	27	Non-ischemic	3	Carvedilol 25 mg	None	Ramipril 2.5 mg
7	58	Female	60	Familial	2	None	None	None
8	35	Male	33	Hypertrophic	3	Carvedilol 50 mg	None	Ramipril 10 mg

^a^LVEF: left ventricular ejection fraction.

^b^HF: heart failure.

^c^NYHA: New York Heart Association.

^d^Drug doses are total daily dose.

